# Growth Differentiation Factor-15 Correlates Inversely with Protease-Activated Receptor-1-Mediated Platelet Reactivity in Patients with Left Ventricular Assist Devices

**DOI:** 10.3390/ph15040484

**Published:** 2022-04-15

**Authors:** Maximilian Tscharre, Franziska Wittmann, Daniela Kitzmantl, Silvia Lee, Beate Eichelberger, Patricia P. Wadowski, Günther Laufer, Dominik Wiedemann, Simon Panzer, Thomas Perkmann, Daniel Zimpfer, Thomas Gremmel

**Affiliations:** 1 Department of Internal Medicine, Cardiology and Nephrology, Landesklinikum Wiener Neustadt, 2700 Wiener Neustadt, Austria; maximilian.tscharre@meduniwien.ac.at; 2 Department of Internal Medicine II, Medical University of Vienna, 1090 Vienna, Austria; daniela.kitzmantl@gmail.com (D.K.); silvia.lee@meduniwien.ac.at (S.L.); patricia.wadowski@meduniwien.ac.at (P.P.W.); 3 Department of Cardiac Surgery, Medical University of Vienna, 1090 Vienna, Austria; franziska.wittmann@meduniwien.ac.at (F.W.); guenther.laufer@meduniwien.ac.at (G.L.); dominik.wiedemann@meduniwien.ac.at (D.W.); daniel.zimpfer@meduniwien.ac.at (D.Z.); 4 Department of Blood Group Serology and Transfusion Medicine, Medical University of Vienna, 1090 Vienna, Austria; thrombozytenlabor@meduniwien.ac.at (B.E.); simon@wsn.at (S.P.); 5 Department of Laboratory Medicine, Medical University of Vienna, 1090 Vienna, Austria; thomas.perkmann@meduniwien.ac.at; 6 Department of Internal Medicine I, Cardiology and Intensive Care Medicine, Landesklinikum Mistelbach-Gänserndorf, 2130 Mistelbach, Austria; 7 Institute of Antithrombotic Therapy in Cardiovascular Disease, Karl Landsteiner Society, 3100 St. Pölten, Austria

**Keywords:** LVAD, GDF-15, multiple electrode aggregometry, GPIIb/IIIa, PAR-1

## Abstract

Growth differentiation factor (GDF)-15 inhibits platelet activation, prevents thrombus formation, and has been linked to bleeding events. This was a prospective study including 51 left-ventricular assist device (LVAD) patients on aspirin and phenprocoumon. Platelet surface expression of activated glycoprotein (GP) IIb/IIIa was assessed by flow cytometry, and platelet aggregation was measured by multiple electrode aggregometry (MEA) in response to arachidonic acid (AA), adenosine diphosphate (ADP), and thrombin receptor-activating peptide (TRAP), a protease-activated-receptor-1 (PAR-1) agonist. GDF-15 was determined with a commercially-available assay. There was a trend towards an inverse correlation of GDF-15 with activated GPIIb/IIIa in response to TRAP (r = −0.275, *p* = 0.0532) but not in response to AA and ADP. Moreover, GDF-15 correlated with MEA TRAP (r = −0.326, *p* = 0.0194), whereas it did not correlate with MEA ADP and MEA AA. In a second step, GDF-15 levels in the fourth quartile were defined as high GDF-15. Patients with high GDF-15 showed significantly lower TRAP-inducible platelet aggregation by MEA compared to patients in the first quartile (63 AU vs. 113 AU, *p* = 0.0065). In conclusion, in LVAD patients receiving state-of-the-art antithrombotic therapy, GDF-15 correlates inversely with residual platelet reactivity via PAR-1.

## 1. Introduction

The introduction of left ventricular assist devices (LVADs) has significantly improved the functional status, quality of life, and prognosis of end-stage heart failure [1].

Due to the activation of the coagulation system in response to artificial surfaces and hemodynamic alterations, LVAD patients are treated with a potent antithrombotic drug regimen comprising of a vitamin-K antagonist (VKA) and aspirin [2,3]. Nevertheless, LVAD patients remain at high risk of thromboembolic events (i.e., pump thrombosis, embolic stroke), and due to the broad inhibition of primary and secondary hemostasis, they are also at high risk of bleeding complications [1].

Growth-differentiation factor (GDF)-15 is a member of the transforming growth factor (TGF)-β family linked with inflammatory processes, metabolic dysfunction, and various cancers [4,5,6,7,8]. In end-stage heart failure patients, GDF-15 levels are significantly elevated but decrease rapidly after LVAD implantation [9]. Nevertheless, GDF-15 levels remain elevated in many LVAD patients, possibly reflecting ongoing remodeling and inflammation [9,10].

Recently, GDF-15 has been demonstrated to partially inhibit platelet integrin activation and to prevent thrombus formation [11,12,13]. In line with these findings, GDF-15 has been associated with bleeding events in several entities, including acute coronary syndromes and atrial fibrillation. Accordingly, GDF-15 has been proposed as a novel biomarker for bleeding complications [14,15,16].

Human platelets can be activated by numerous agonists via different pathways [17]. In particular, arachidonic acid (AA), adenosine diphosphate (ADP), and thrombin are strong platelet agonists [18]. AA exerts its effect by activating the generation of thromboxane A2, ADP activates two G-protein coupled receptors (P2Y1 and P2Y12) on human platelets, and thrombin acts as an agonist for protease-activated receptor (PAR)-1 and -4 [17,18]. These pathways are of major interest, as their inhibition by available antiplatelet drugs has been demonstrated to reduce ischemic events. However, the latter is achieved at the expense of an increased risk of bleeding complications [18]. P-selectin expression and activated GPIIb/IIIa are sensitive parameters of platelet activation and have been associated with ischemic events [19]. After activation, platelets express P-selectin on their surface, which facilitates the generation of monocyte-platelet aggregates [20,21]. Activated GPIIb/IIIa binds to its ligand fibrinogen and thereby enables the interaction with coagulation factors and other platelets [22]. Through the interaction of platelets with coagulation factors, leukocytes, and other platelets, platelet activation induces a prothrombotic and proinflammatory milieu.

Since balancing the risk of bleeding events and thromboembolic complications remains a major challenge in the management of LVAD patients, we investigated the role of GDF-15 in on-treatment platelet reactivity and bleeding complications in a cohort of stable LVAD patients treated with aspirin and a VKA.

## 2. Results

**Figure 1:**
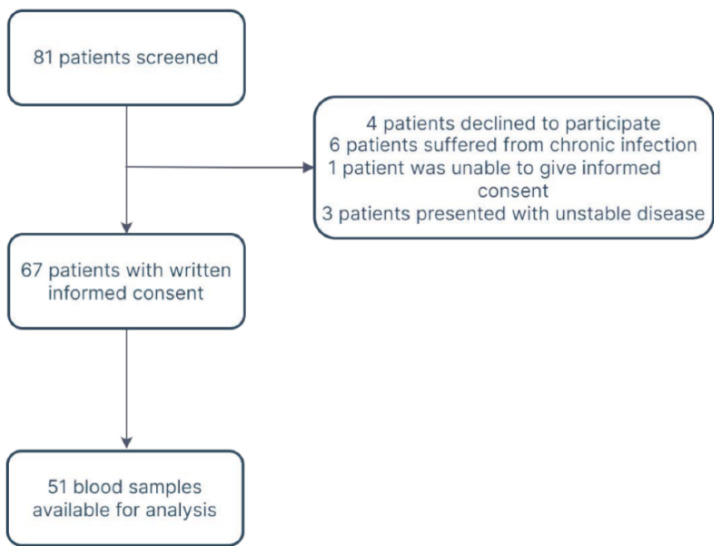
Flow diagram.

In total, 51 patients were available for analysis (Figure 1, Supplementary Table S1).

The median age was 62 (IQR 55–69) years, and 46 (90.2%) patients were male. HVAD was implanted in 15 (29.4%), HM2 in 2 (3.9%), and HM3 in 34 (66.7%) patients. The median time interval from LVAD implantation to study inclusion was 370 days (IQR 174–906). Median GDF-15 levels were 2181 (IQR 1336–3231) pg/mL. Clinical, laboratory, and procedural characteristics for the entire cohort are presented in Table 1.

**Figure 2:**
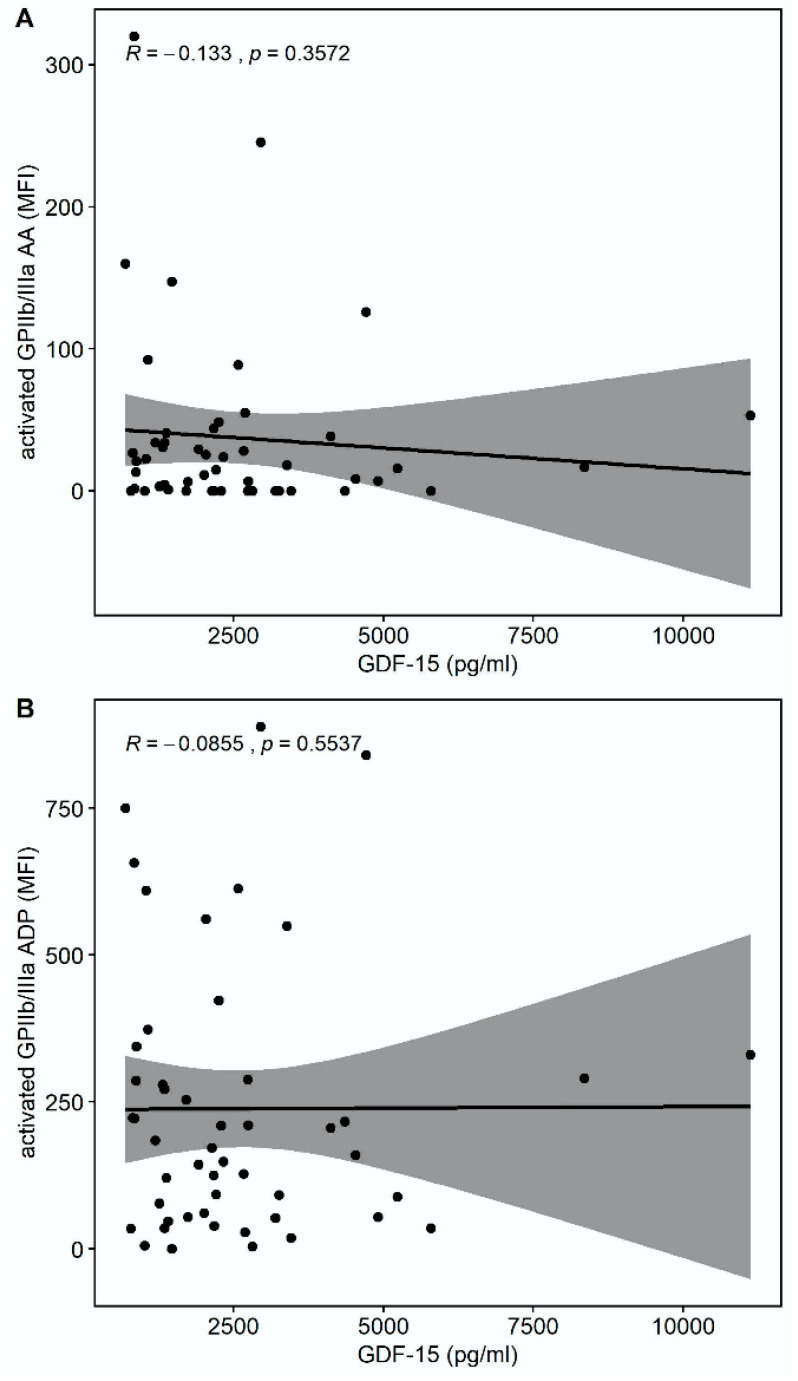

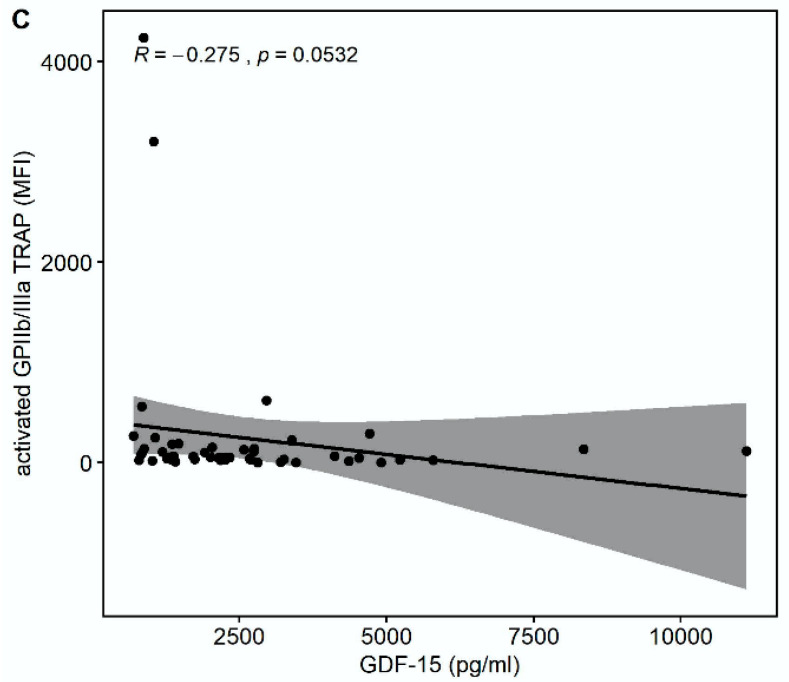
Correlations of GDF-15 with the platelet surface expression of activated glycoprotein (GP) IIb/IIIa. (**A**) Scatter plot showing GDF-15 (*x*-axis) versus activated GPIIb/IIIa in response to arachidonic acid (AA) (*y*-axis). (**B**) Scatter plot showing GDF-15 (*x*-axis) versus activated GPIIb/IIIa in response to adenosine diphosphate (ADP) (*y*-axis). (**C**) Scatter plot showing GDF-15 (*x*-axis) versus activated GPIIb/IIIa in response to thrombin receptor-activating peptide (TRAP) (*y*-axis).

There was a trend towards a correlation of GDF-15 with the platelet surface expression of activated glycoprotein (GP) GPIIb/IIIa in response to thrombin receptor-activating peptide (TRAP) (r = −0.275, *p* = 0.0532) but not in response to arachidonic acid (AA) (r = −0.133, *p* = 0.3572) or adenosine-diphosphate (ADP) (r = −0.0855, *p* = 0.5537), as shown in Figure 2.

**Figure 3:**
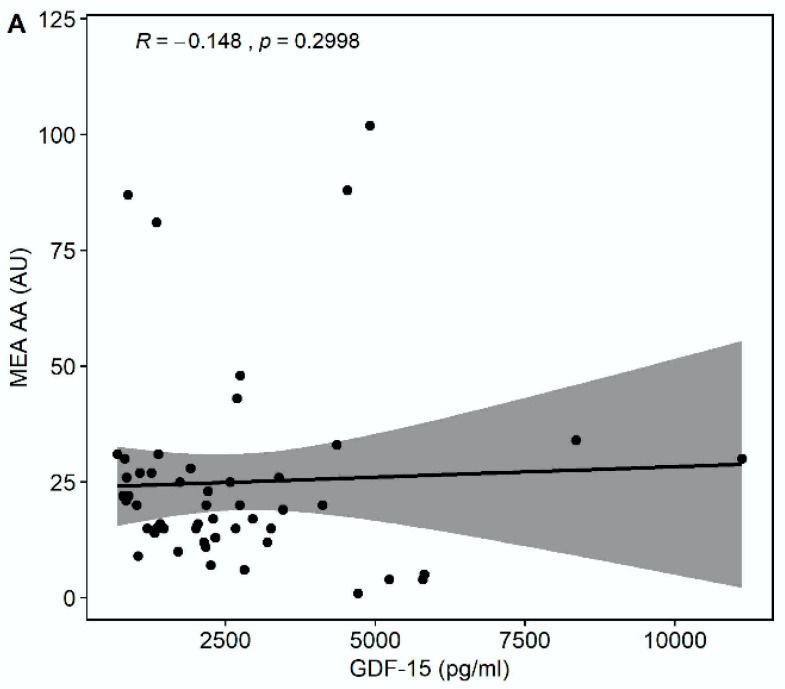

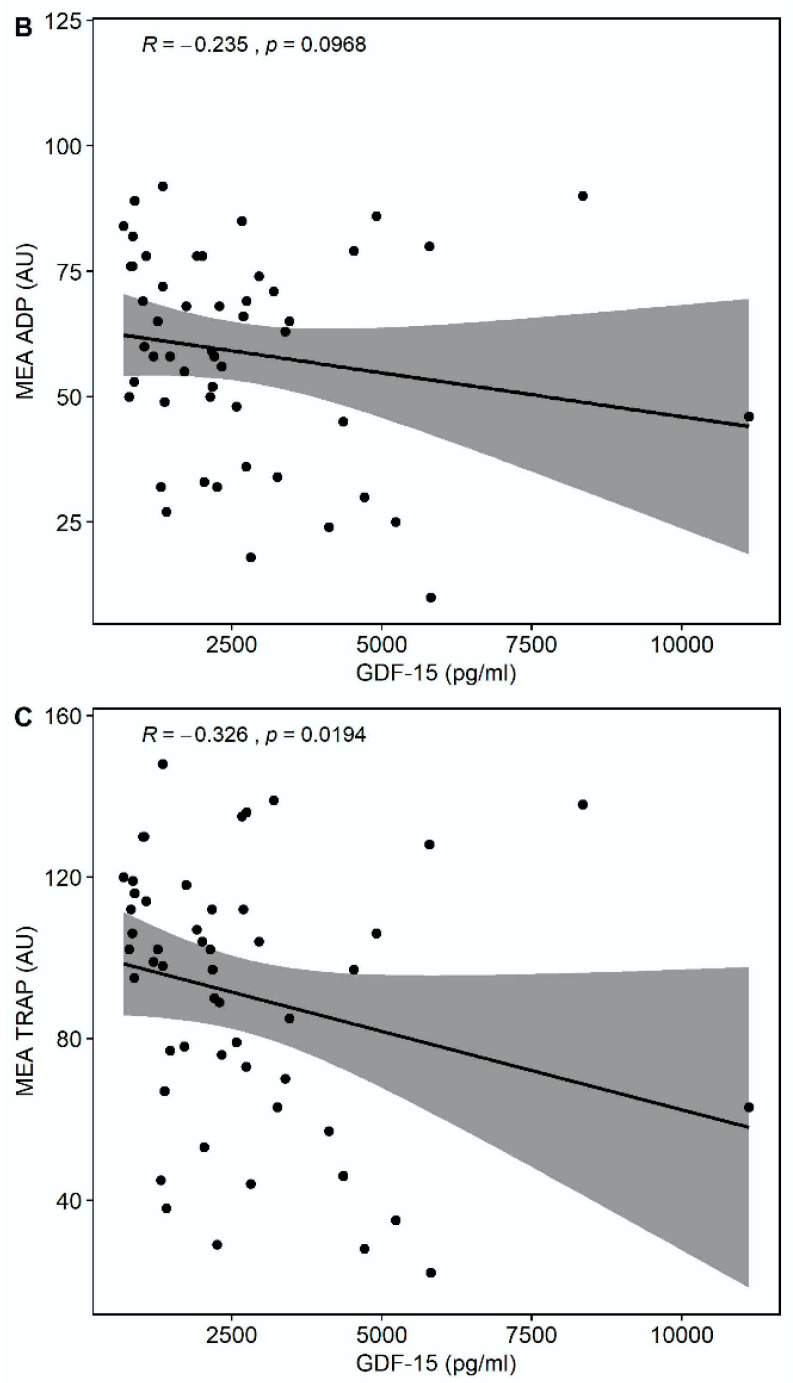
Correlations of GDF-15 with platelet aggregation by multiple electrode aggregometry (MEA). (**A**) Scatter plot showing GDF-15 (*x*-axis) versus arachidonic acid (AA)-inducible platelet aggregation by MEA (*y*-axis). (**B**) Scatter plot showing GDF-15 (*x*-axis) versus adenosine diphosphate (ADP)-inducible platelet aggregation by MEA (*y*-axis). (**C**) Scatter plot showing GDF-15 (*x*-axis) versus thrombin receptor-activating peptide (TRAP)-inducible platelet aggregation by MEA (*y*-axis).

Median multiple electrode aggregation (MEA) AA was 20 AU (IQR 14–28), median MEA ADP was 60 AU (IQR 48–74), and median MEA TRAP was 98 AU (IQR 67–114). GDF-15 correlated inversely with MEA TRAP (r = −0.326, *p* = 0.0194), whereas the correlation of GDF-15 with MEA ADP (r = −0.235, *p* = 0.0968) and MEA AA (r = −0.148, *p* = 0.2998) did not reach statistical significance (Figure 3). GDF-15 did not correlate with soluble P-selectin (r = −0.062, *p* = 0.626).

In a second step, GDF-15 levels in the fourth quartile were defined as high GDF-15, and GDF-15 levels in the first quartile were defined as low GDF-15. Accordingly, 12 patients (23.5%) had high GDF-15, and 13 patients (25.5%) had low GDF-15. Baseline characteristics according to the quartiles of GDF-15 are shown in Table 2.

**Figure 4:**
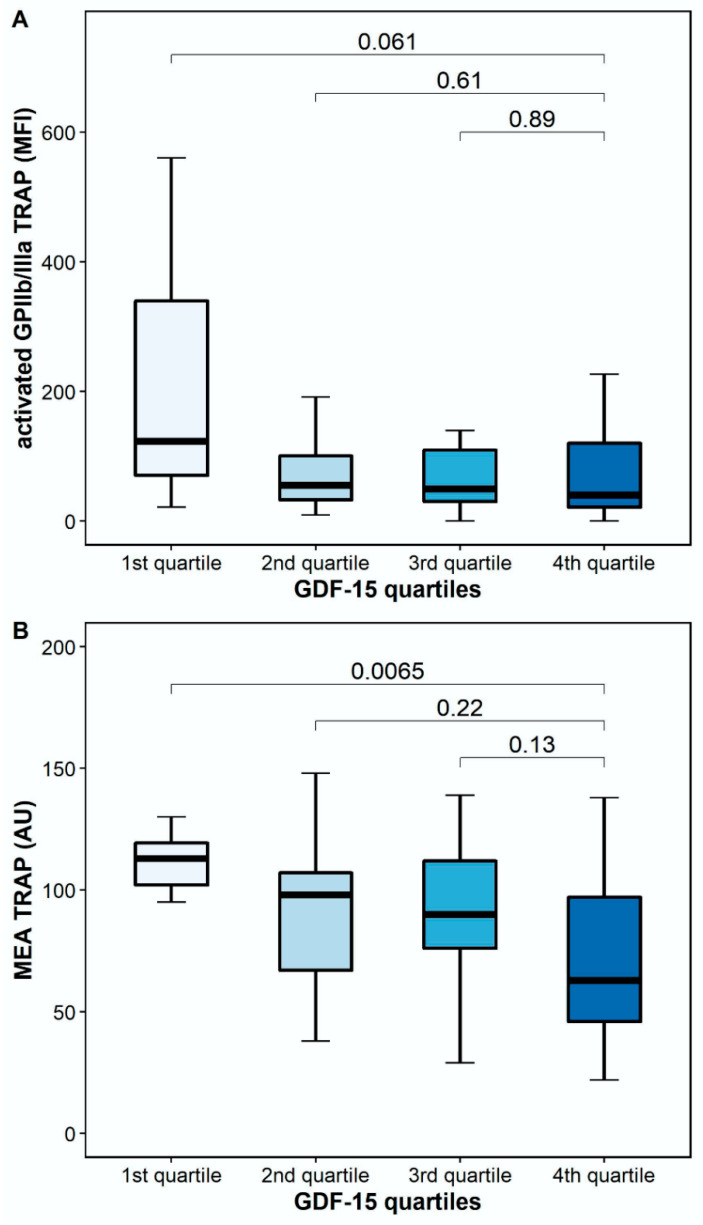
Platelet reactivity in response to thrombin receptor-activating peptide (TRAP) according to GDF-15 quartiles. (**A**) Activated GPIIb/IIIa in response to TRAP according to GDF-15 quartiles. (**B**) TRAP-inducible platelet aggregation by multiple electrode aggregometry according to GDF-15 quartiles. The boundaries of the box show the lower and upper quartile of data, and the line inside the box represents the median. Whiskers were drawn from the edge of the box to the highest and lowest values that are outside the box but within 1.5 times the box length. The outliers are not presented.

The platelet surface expression of activated GPIIb/IIIa in response to TRAP was numerically lower, but it did not differ significantly between patients with high and low GDF-15 (40.5 MFI [IQR 17.6–128.6] vs. 123.6 MFI [IQR 48.4–487.1], *p* = 0.061). However, TRAP-inducible platelet aggregation by MEA was significantly lower in patients with high GDF-15 compared to patients with low GDF-15 (63 AU [IQR 41–102] vs. 113 AU [IQR 102–120], *p* = 0.0065; Figure 4).

**Figure 5:**
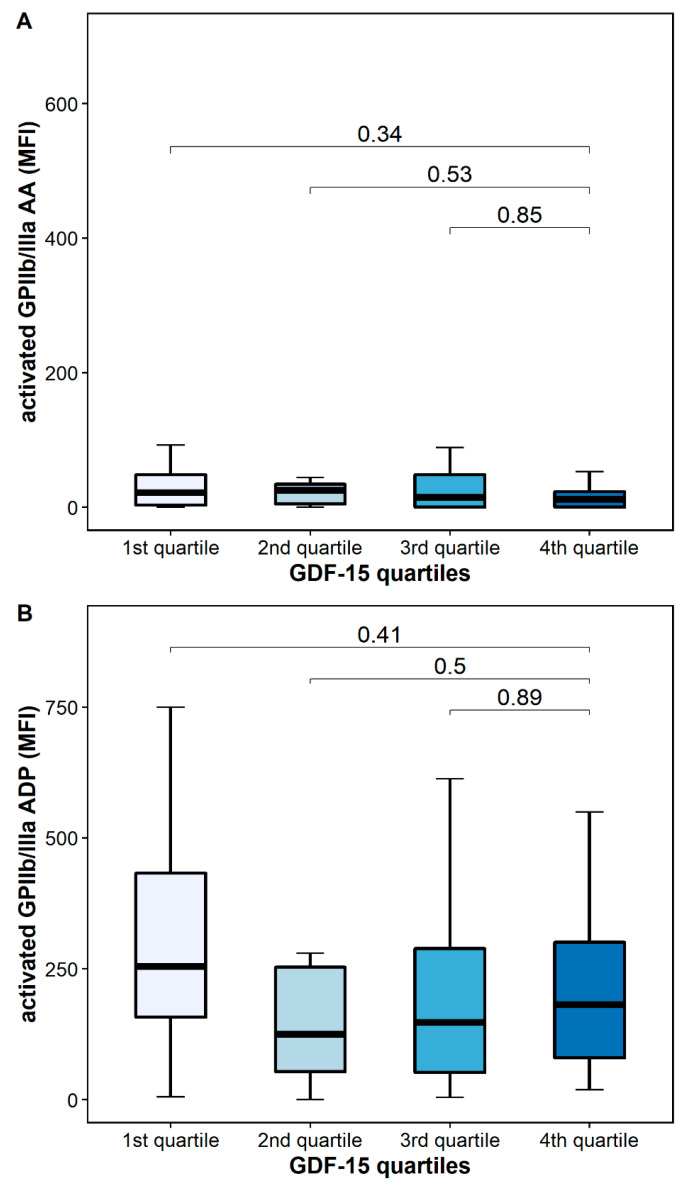
Platelet activation according to GDF-15 quartiles. (**A**) Activated GPIIb/IIIa in response to arachidonic acid (AA). (**B**) Activated GPIIb/IIIa in response to adenosine diphosphate (ADP). The boundaries of the box show the lower and upper quartile of data, and the line inside the box represents the median. Whiskers were drawn from the edge of the box to the highest and lowest values that are outside the box but within 1.5 times the box length.

The platelet surface expression of activated GPIIb/IIIa in response to AA was similar in patients with high and low GDF-15 (12 MFI [IQR 0–33] vs. 24 MFI [IQR 10–109], *p* = 0.34), whereas the platelet surface expression of activated GPIIb/IIIa in response to ADP was numerically decreased in patients with high GDF-15 without reaching statistical significance [182 MFI [IQR 63–320] vs. 315 MFI [213–621], *p* = 0.41] (Figure 5).

**Figure 6:**
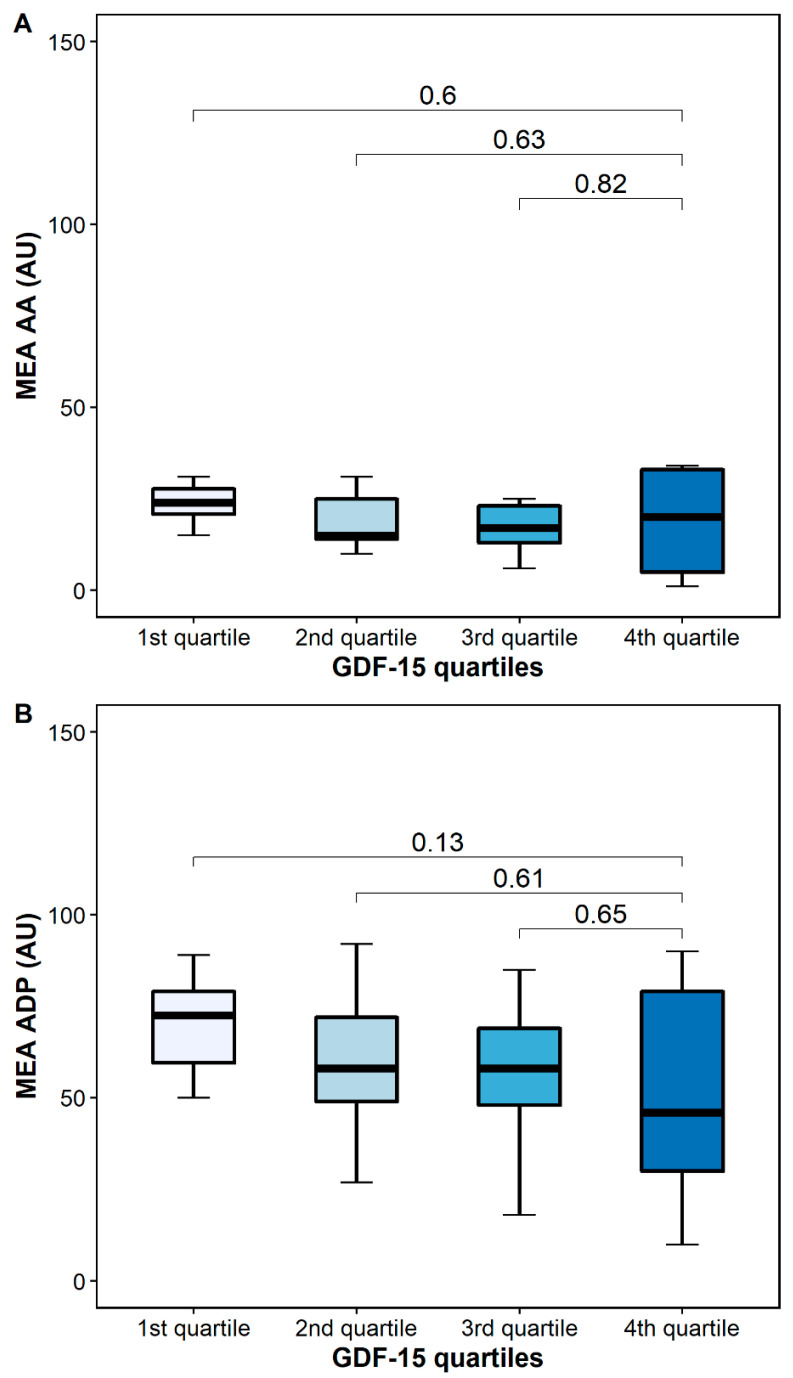
Platelet aggregation by multiple electrode aggregometry (MEA) according to GDF-15 quartiles. (**A**) Arachidonic acid-inducible platelet aggregation by multiple electrode aggregometry. (**B**) Adenosine diphosphate (ADP)-inducible platelet aggregation by multiple electrode aggregometry. The boundaries of the box show the lower and upper quartiles of data, and the line inside the box represents the median. Whiskers were drawn from the edge of the box to the highest and lowest values that are outside the box but within 1.5 times the box length.

AA-inducible platelet aggregation by MEA was similar in patients with high and low GDF-15 (24 AU [IQR 20–30] vs. 20 [5–34], *p* = 0.6), whereas ADP-inducible platelet aggregation by MEA was numerically higher in patients with low GDF-15 (76 AU (54–82] vs. 46 AU [27–80], *p* = 0.13) (Figure 6).

Follow-up was available for all patients. Six patients (11.7%) experienced bleeding complications during the follow-up. Five patients suffered severe gastrointestinal bleeding requiring blood transfusions (GDF-15 levels: 2172 pg/mL, 2255 pg/mL, 2297 pg/mL, 2333 pg/mL, and 8347 pg/mL), and one patient suffered macrohematuria due to urothelial carcinoma, not requiring a blood transfusion (GDF-15 level: 851 pg/mL). Patients experiencing bleeding events were numerically older (68 years [IQR 64–70 years] vs. 60 years [IQR 52–70 years], *p* = 0.146), while the other baseline characteristics did not differ significantly between patients with and without bleeding complications. One patient received triple therapy including clopidogrel.

## 3. Discussion

To the best of our knowledge, our study is the first to associate GDF-15 plasma levels with on-treatment residual platelet reactivity in LVAD patients treated with aspirin and a VKA. In our cohort, GDF-15 correlated inversely with PAR-1-mediated platelet aggregation. Moreover, patients with high GDF-15 had a numerically lower platelet surface expression of activated GPIIb/IIIa in response to TRAP and significantly lower TRAP-inducible platelet aggregation by MEA, as compared to patients with low GDF-15 levels.

Activated GPIIb/IIIa is the most abundant receptor on human platelets and acts as a binding site for fibrinogen and the von Willebrand factor, thereby facilitating the interaction of platelets with coagulation factors and other platelets, ultimately resulting in platelet aggregation [18]. Accordingly, activated GPIIb/IIIa is a sensitive marker of platelet activation. Recently, platelet surface expression of activated GPIIb/IIIa has been associated with ischemic outcomes in patients with atherosclerotic disease [19]. MEA is a fast, easily-applicable, and highly-standardized platelet function test that estimates the agonist-inducible platelet aggregation as an increase in electrical impedance between two electrodes [23]. In several observational investigations, on-treatment platelet reactivity, as assessed by MEA, repeatedly predicted adverse outcomes in patients with atherosclerotic disease. While high on-treatment residual platelet reactivity has been associated with thrombotic events in patients undergoing percutaneous coronary interventions, a strong anti-aggregatory response resulting in low on-treatment residual platelet reactivity was linked to an increase in bleeding events [24,25,26,27]. Platelet activation and aggregation are initiated by several agonists via different pathways. Only a few of these pathways can be inhibited by currently-available antiplatelet agents [18,28]. We chose to investigate platelet reactivity in response to AA, ADP, and TRAP, as these agonists are pivotal for platelet activation, and their pathways are all major targets of orally-available antiplatelet agents.

Kempf et al. demonstrated that GDF-15 inhibits myeloid cell recruitment into infarcted myocardium by inhibiting chemokine-triggered adhesion and trans-endothelial infiltration [8]. Mechanistically, GDF-15 reduces the conformational activation and clustering of β2-integrins by activating Cdc42 and inhibiting the activation of Rap1, a GTPase that mediates integrin activation [8,29]. Likewise, GDF-15 has also been demonstrated to interfere with platelet integrin activation and platelet aggregation [11]. By inhibiting Rap1, GDF-15 partially impedes agonist-induced platelet αIIbβ3 integrin (the GPIIb/IIIa complex) activation in vitro and prevents thrombus formation in vivo in mice [11]. Of note, platelet P-selectin expression and dense granule secretion was not affected by GDF-15 [11]. In line with these results, we found no correlation of soluble P-selectin with GDF-15 levels in our cohort.

GDF-15 has been associated with bleeding complications in numerous studies, including patients with coronary artery disease on dual antiplatelet therapy (DAPT) and patients with atrial fibrillation on oral anticoagulation [14,15,30]. Consequently, GDF-15 has been proposed as a novel biomarker for bleeding events and has been included in the novel ABC-bleeding risk score for patients with atrial fibrillation [16]. Since only six patients in our study developed bleeding complications, we cannot draw definitive conclusions on the applicability of GDF-15 as risk marker for bleeding in LVAD patients. However, we detected an inverse association of GDF-15 with the platelet surface expression of activated GPIIb/IIIa via PAR-1 and with PAR-1-mediated platelet reactivity by MEA. PAR-1 is activated by thrombin, a strong endogenous platelet agonist, and, in conjunction with PAR-4, leads to ADP release, promoting the further recruitment, adhesion, and aggregation of activated platelets [31]. We recently demonstrated that PAR-1–mediated platelet reactivity is preserved in most patients with atherosclerotic disease receiving DAPT, even in patients receiving the more potent P2Y12 inhibitors prasugrel and ticagrelor [32,33]. Furthermore, we showed that residual PAR-1-mediated platelet activation is associated with ischemic events in patients undergoing peripheral angioplasty with stenting [19]. Accordingly, platelet activation via PAR-1 may represent a prothrombotic marker that is independent of conventional DAPT with aspirin and a P2Y12 inhibitor [19]. In line with these findings, the selective inhibition of PAR-1 with vorapaxar resulted in a significant decrease in thrombotic events in patients with coronary artery disease on DAPT but also led to an increase in bleeding complications, including cerebral hemorrhage [34]. The latter suggests that low PAR-1-mediated platelet reactivity might be associated with an increased bleeding risk.

In our study, GDF-15 correlated only with TRAP-inducible but not with AA- or ADP-inducible platelet activation and aggregation. However, these findings are not surprising as all patients in our cohort received aspirin, which is a reliable inhibitor of AA-inducible platelet aggregation by irreversibly blocking cyclooxygenase-1 [18]. This is supported by our data, in which platelet activation and aggregation in response to AA was inhibited potently and similarly across GDF-15 quartiles (Figure 5 and Figure 6). Furthermore, former studies have demonstrated that aspirin also partially inhibits platelet activation and aggregation via the ADP pathway [35]. This fact potentially explains the linear but not significant associations of platelet activation and aggregation in response to AA and ADP seen across GDF-15 quartiles (Figure 5 and Figure 6).

Balancing antithrombotic treatment in patients with LVAD remains a major challenge. Based on our findings, one may speculate that GDF-15 could help to identify LVAD patients at high risk of bleeding complications who benefit from a less aggressive antithrombotic treatment regimen. GDF-15 might become part of a biomarker-based bleeding risk score in LVAD patients, similar to the ABC-bleeding score in patients with atrial fibrillation [36]. However, the introduction of risk scores in routine clinical practice demands derivation and validation cohort studies that include considerable patient numbers. Therefore, in order to establish a meaningful bleeding risk score that includes GDF-15 in LVAD patients, further accurate planning and a joint effort of numerous LVAD centers will be needed. On the other hand, GDF-15 might help to identify patients at high risk of thrombosis who may benefit from additional antithrombotic therapy, i.e., a PAR-1 antagonist. Higher GDF-15 levels have been associated with bleeding risk particularly in patients with atrial fibrillation and after acute coronary syndromes. However, a recently published observational trial also linked higher GDF-15 levels with incidental intracerebral and subarachnoid hemorrhage in the general population, independently of other known risk factors [37]. Accordingly, GDF-15 may constitute a marker of overall bleeding hazard and might be used to estimate bleeding risk in patients with end-stage heart failure or even perioperatively. However, no data supporting this assumption have been published so far, and further clinical studies are needed to draw definitive conclusions.

### Limitations

The present study has the following limitations: First, our data derived from a single center. Second, different aspirin dosages were used for patients with HVAD, HM2, and HM3. However, in previous studies, aspirin dosages down to 75 mg daily have been shown to completely inhibit platelet thromboxane production. Therefore, a relevant impact on our results is unlikely [38,39]. Another limitation is our sample size. Finally, due to the low number of adverse events, no outcome analyses were performed.

We exclusively included patients in a stable clinical condition to avoid any influence of inflammation, bleeding, or acute thrombosis on platelet function. Accordingly, the association of GDF-15 with PAR-1 mediated platelet reactivity may differ in unstable patients. All patients received the best medical treatment and antithrombotic therapy with aspirin and phenprocoumon. Therefore, it is unlikely that pre-analytical differences or differences in cardiovascular risk factor management influenced our observations.

## 4. Materials and Methods

We conducted a prospective single-center study including 51 patients with LVADs (Medtronic/Heartware HVAD (HVAD), Thoratec HeartMate II (HM2), or Thoratec HeartMate 3 (HM3)) for the treatment of end-stage heart failure as a bridge to transplantation, bridge to candidacy, or destination therapy between January 2018 and October 2020. Patients were enrolled at the outpatient department of the Department of Cardiac Surgery of the Medical University of Vienna. LVAD implantation was performed ≥3 months before enrollment, and all patients were in a stable clinical condition. All patients received long-term antithrombotic therapy with aspirin (HVAD: 200 mg daily; HM2 and HM3: 100 mg daily) and the VKA phenprocoumon (target international normalized ratio (INR) 2.0–3.0) [3].

Our cohort is the first to investigate the relationship of platelet function and GDF-15 in patients receiving antiplatelet therapy. Therefore, no meaningful sample size calculation was possible, and the study must be regarded as observational and exploratory in nature.

The exclusion criteria were known aspirin intolerance (allergic reactions, history of bleeding events), a family or personal history of bleeding disorders, acute or chronic infection, malignant paraproteinemia, myeloproliferative disorders, severe hepatic failure, a major surgical procedure within one week before enrollment, known qualitative defects in thrombocyte function, a platelet count <100.000 or >450.000/μL, and a hematocrit <30%.

The study was approved by the Ethics Committee of the Medical University of Vienna (1765/2017, 11 September 2017) in accordance with the Declaration of Helsinki, and written informed consent was obtained from all study participants.

### 4.1. Blood Sampling

Blood was drawn by aseptic venipuncture from an antecubital vein using a 21-gauge butterfly needle (0.8 × 19 mm; Greiner Bio-One, Kremsmünster, Austria) as previously described [40]. To avoid procedural deviations, all blood samples were taken by the same physician applying a light tourniquet that was immediately released, and the samples were mixed by gently inverting the tubes.

### 4.2. Platelet Surface Expression of Activated Glycoprotein IIb/IIIa

The binding of the monoclonal antibody PAC-1 to activated GPIIb/IIIa was determined in citrate-anticoagulated blood, as previously described [40]. In brief, whole blood was diluted in phosphate-buffered saline to obtain 20 × 10^3^/μL platelets in 20 μL and incubated for 10 min with the platelet-specific monoclonal antibody anti-CD42b (clone HIP1, allophycocyanin labeled; Becton Dickinson (BD), San Jose, CA, USA). After in vitro exposure to suboptimal concentrations of arachidonic acid (AA; final concentration 80 µM; Roche Diagnostics, Mannheim, Germany), adenosine diphosphate (ADP; final concentration 1 μM; Roche Diagnostics, Mannheim, Germany) or thrombin receptor-activating peptide (TRAP; a protease-activated receptor [PAR]-1 agonist; final concentration 14.25 µM; Bachem, Bubendorf, Switzerland), each 10 μL for 10 min, samples were incubated for another 10 min with a mixture of antibodies against activated GPIIb/IIIa (the monoclonal antibody PAC-1-fluorescein (BD)). Isotype-matched control antibodies (BD) were used for the determination of non-specific binding. After 10 min of incubation in the dark, the reaction was stopped by adding 500 μL of phosphate-buffered saline, and samples were acquired on a FACSCanto II flow cytometer (BD). At acquisition, the platelet population was identified by its characteristics in the forward scatter versus side scatter plot. A total of 10,000 events were acquired within this gate. This population was further identified by platelets stained with the platelet-specific monoclonal antibody anti-CD42b versus side scatter. The binding of the antibodies against activated GPIIb/IIIa was determined in histograms for PAC-1. The MFI based on all events was used for statistical calculations.

### 4.3. Multiple Electrode Platelet Aggregometry (MEA)

Whole blood impedance aggregometry was performed with the Multiplate analyzer (Roche Diagnostics, Mannheim, Germany) as previously described [40]. After dilution (1:2 with 0.9% sodium chloride solution) of hirudin-anticoagulated whole blood and stirring in the test cuvettes for 3 min at 37 °C, AA (0.5 mM), ADP (6.4 μM), or TRAP (32 µM; all from Roche Diagnostics, Mannheim, Germany) was added, and aggregation was continuously recorded for six minutes. The adhesion of activated platelets to the electrodes led to an increase of impedance, which was detected for each sensor unit separately and transformed to aggregation units (AU) that were plotted against time. The AU at 6 min were used for all calculations. One AU corresponds to 10 AU * min (area under the curve of AU).

### 4.4. Growth Differentiation Factor 15 (GDF-15)

GDF-15 was measured on a cobas^®^ e602 modular analyzer (Roche Diagnostics, Mannheim, Germany) according to the manufacturer’s instructions using the CE-marked Roche Elecsys^®^ GDF-15 electrochemiluminescence sandwich immunoassay (ECLIA) (Roche Diagnostics, Rotkreuz, Switzerland).

### 4.5. Soluble P-Selectin

Soluble P-selectin was measured according to the manufacturer’s instructions, as described previously (Human sP-Selectin Immunoassay, R&D Systems, Minneapolis, MN, USA) [40].

### 4.6. Clinical Endpoint

The composite of major and minor bleedings, according to the definition of the International Society on Thrombosis and Haemostasis (ISTH), was defined as the clinical endpoint [41]. Clinical follow-up was assessed for all patients at the outpatient department of the Department of Cardiac Surgery at the Medical University of Vienna, where all LVAD patients have regular visits every three months.

### 4.7. Statistics

All continuous variables are expressed as median (interquartile range [IQR]). Categorical variables are given as number (%). Continuous variables were compared by Mann-Whitney-U-test or Kruskal-Wallis-H-test for independent samples. χ^2^-tests were performed for comparison of categorical variables. The Spearman rank correlation was used to test for correlation. All statistical tests were 2-tailed, and a *p*-value < 0.05 was required for statistical significance. All statistical analyses and figures were performed with R 4.1.1 (R: A language and environment for statistical computing, R Foundation for Statistical Computing, Vienna, Austria) and SPSS 24.0 (Armonk, NY, USA).

## 5. Conclusions

In LVAD patients receiving state-of-the-art antithrombotic therapy, GDF-15 was inversely correlated with on-treatment residual platelet reactivity via PAR-1. Further clinical trials are needed to investigate if GDF-15 might help to identify LVAD patients at risk of bleeding and to guide antithrombotic therapy.

## Figures and Tables

**Table 1:** Baseline characteristics.

	Entire Cohort
	(*n* = 51)
GDF-15, pg/mL	2181 [1336–3231]
Age, years	62 [55–69]
Sex, No. (%):	
Female patients	5 (9.8%)
Male patients	46 (90.2%)
Body mass index, kg/m^2^	29.4 [25.9–31.8]
Type of cardiomyopathy, No. (%):	
Ischemic CMP	38 (74.5%)
Dilatative CMP	13 (25.5%)
Ventricular assist device, No. (%):	
HVAD	15 (29.4%)
HM2	2 (3.92%)
HM3	34 (66.7%)
Flow min^−1^	4.90 [4.30–5.18]
Speed RPM	5350 [2925–5700]
History of arterial hypertension, No. (%)	29 (56.9%)
Hyperlipidemia, No. (%)	23 (45.1%)
Diabetes mellitus, No. (%)	10 (19.6%)
Atrial fibrillation, No. (%)	20 (39.2%)
Cerebral artery disease, No. (%)	2 (3.92%)
Peripheral artery disease, No. (%)	2 (3.92%)
Hemoglobin, g/dL	13.3 [11.7–14.4]
Thrombocytes, G/L	222 [178–265]
White blood cell count, G/L	8.28 [6.25–9.73]
Creatinine, mg/dL	1.18 [1.02–1.64]
INR	2.4 [2.1–2.7]
proBNP, pg/mL	1022 [567–1691]
High sensitivity C-reactive protein, mg/dL	0.27 [0.13–0.59]
Vitamin K antagonist, No. (%)	51 (100%)
Aspirin, No. (%)	51 (100%)
Clopidogrel, No. (%)	2 (3.9%)
Statin, No. (%)	34 (69.4%)
ACE-I or ARB, No. (%)	36 (70.6%)
MRA, No. (%)	28 (54.9%)
Furosemide, No. (%)	45 (88.2%)
Beta blocker, No. (%)	34 (68.0%)

Continuous data are shown as median [interquartile range]. Dichotomous data are shown as *n* (%). ACE-I = angiotensin-converting enzyme inhibitor; ARB = angiotensin-receptor blocker; CMP = cardiomyopathy; INR = international normalized ratio; MRA = mineralocorticoid receptor antagonist; proBNP = pro brain-natriuretic peptide; RPM = rounds per minute.

**Table 2:** Baseline characteristics according to the quartiles of GDF-15.

	1st Quartile	2nd Quartile	3rd Quartile	4th Quartile	*p*-Value
	*n* = 13	*n* = 13	*n* = 13	*n* = 12	
Age, years	56 [52–68]	59 [56–64]	61 [56–71]	69 [61–71]	0.384
Sex, No. (%):					0.536
Female patients	0 (0.0%)	2 (15.4%)	1 (7.7%)	2 (16.7%)	0.536
Male patients	13 (100%)	11 (84.6%)	12 (92.3%)	10 (83.3%)	
Ventricular assist device, No. (%):					
HVAD	5 (38.5%)	1 (7.7%)	5 (38.5%)	4 (33.3%)	0.476
HM2	1 (7.7%)	1 (7.7%)	0 (0.0%)	0 (0.0%)	
HM3	7 (53.8%)	11 (84.6%)	8 (61.5%)	8 (66.7%)	
Body mass index, kg/m^2^	31.0 [28.2–35.1]	28.4 [25.6–30.4]	31.0 [28.8–32.8]	29.0 [27.0–30.3]	0.279
Type of cardiomyopathy, No. (%):					
Ischemic CMP	10 (76.9%)	9 (69.2%)	8 (61.5%)	11 (91.7%)	0.420
Dilatative CMP	3 (23.1%)	4 (30.8%)	5 (38.5%)	1 (8.33%)	
Flow, min-1	5.3 [5.0–5.5]	4.9 [4.0–5.0]	4.7 [4.5–4.9]	4.7 [4.2–5.1]	0.089
Speed RPM	4200 [2850–5750]	5700 [5600–6000]	5200 [2775–5325]	5200 [2810–5450]	0.024
History of arterial hypertension, No. (%)	7 (53.8%)	9 (69.2%)	7 (53.8%)	6 (50.0%)	0.768
Hyperlipidemia, No. (%)	4 (30.8%)	8 (61.5%)	7 (53.8%)	4 (33.3%)	0.312
Atrial fibrillation, No. (%)	3 (23.1%)	5 (38.5%)	7 (53.8%)	5 (41.7%)	0.497
Cerebral artery disease, No. (%)	0 (0.0%)	0 (0.0%)	0 (0.0%)	2 (16.7%)	0.040
Peripheral artery disease, No. (%)	0 (0.0%)	1 (7.7%)	1 (7.7%)	0 (0.0%)	1.000
Hemoglobin, g/dL	14.4 [13.2–16.5]	13.6 [12.0–14.1]	13.5 [12.2–14.4]	11.7 [10.8–12.6]	0.010
Thrombocytes, G/L	250 [195–278]	222 [188–244]	225 [183–279]	177 [154–249]	0.551
White blood cell count, G/L	7.1 [6.4–8.5]	8.6 [5.6–10.3]	8.0 [5.9–9.8]	8.7 [7.6–10.1]	0.413
Creatinine, mg/dL	1.0 [0.9–1.1]	1.1 [1.1–1.4]	1.3 [1.0–1.6]	1.7 [1.1–2.1]	0.008
INR	2.5 [2.3–2.6]	2.5 [2.2–2.7]	2.2 [2.1–2.7]	2.2 [2.1–2.4]	0.328
High sensitivity C-reactive protein, mg/L	0.2 [0.1–0.3]	0.2 [0.1–0.4]	0.4 [0.2–0.7]	0.6 [0.2–2.2]	0.050
Vitamin K antagonist, No. (%)	13 (100%)	13 (100%)	13 (100%)	12 (100%)	.
Aspirin, No. (%)	13 (100%)	13 (100%)	13 (100%)	12 (100%)	.
Clopidogrel, No. (%)	0 (0.0%)	0 (0.0%)	2 (15.4%)	0 (0.0%)	0.219
ACE-I or ARB, No. (%)	8 (61.5%)	10 (76.9%)	9 (69.2%)	6 (50.0%)	0.570
Furosemide, No. (%)	10 (76.9%)	13 (100%)	10 (76.9%)	12 (100%)	0.145
Statin, No. (%)	7 (58.3%)	10 (76.9%)	8 (61.5%)	9 (81.8%)	0.562

Continuous data are shown as median [interquartile range]. Dichotomous data are shown as *n* (%). ACE-I = angiotensin-converting enzyme inhibitor; ARB = angiotensin-receptor blocker; CMP = cardiomyopathy; INR = international normalized ratio; MRA = mineralocorticoid receptor antagonist; proBNP = pro brain-natriuretic peptide; RPM = rounds per minute.

## Data Availability

Data is contained within the article and supplementary material.

## Supplementary Materials

The following supporting information can be downloaded at: https://www.mdpi.com/article/10.3390/ph15040484/s1, Table S1: Baseline characteristics comparing patients included in the final analysis and patients without available plasma sample.

## References

[1] Kirklin J.K., Naftel D.C., Pagani F.D., Kormos R.L., Stevenson L.W., Blume E.D., Myers S.L., Miller M.A., Baldwin J.T., Young J.B. (2015). Seventh INTERMACS Annual Report: 15,000 Patients and Counting. J. Heart Lung Transplant..

[2] Maltais S., Kilic A., Nathan S., Keebler M., Emani S., Ransom J., Katz J.N., Sheridan B., Brieke A., Egnaczyk G. (2017). PREVENtion of HeartMate II Pump Thrombosis Through Clinical Management: The PREVENT Multi-Center Study. J. Heart Lung Transplant..

[3] Potapov E.V., Antonides C., Crespo-Leiro M.G., Combes A., Färber G., Hannan M.M., Kukucka M., De Jonge N., Loforte A., Lund L.H. (2019). 2019 EACTS Expert Consensus on Long-Term Mechanical Circulatory Support. Eur. J. Cardio Thorac. Surg..

[4] Bootcov M.R., Bauskin A.R., Valenzuela S.M., Moore A.G., Bansal M., He X.Y., Zhang H.P., Donnellan M., Mahler S., Pryor K. (1997). MIC-1, a Novel Macrophage Inhibitory Cytokine, Is a Divergent Member of the TGF-β Superfamily. Proc. Natl. Acad. Sci. USA.

[5] Farhan S., Freynhofer M.K., Brozovic I., Bruno V., Vogel B., Tentzeris I., Baumgartner-Parzer S., Huber K., Kautzky-Willer A. (2016). Determinants of Growth Differentiation Factor 15 in Patients with Stable and Acute Coronary Artery Disease. A Prospective Observational Study. Cardiovasc. Diabetol..

[6] Mullican S.E., Lin-Schmidt X., Chin C.-N., Chavez J.A., Furman J.L., Armstrong A.A., Beck S.C., South V.J., Dinh T.Q., Cash-Mason T.D. (2017). GFRAL Is the Receptor for GDF15 and the Ligand Promotes Weight Loss in Mice and Nonhuman Primates. Nat. Med..

[7] Suriben R., Chen M., Higbee J., Oeffinger J., Ventura R., Li B., Mondal K., Gao Z., Ayupova D., Taskar P. (2020). Antibody-Mediated Inhibition of GDF15–GFRAL Activity Reverses Cancer Cachexia in Mice. Nat. Med..

[8] Kempf T., Zarbock A., Widera C., Butz S., Stadtmann A., Rossaint J., Bolomini-Vittori M., Korf-Klingebiel M., Napp L.C., Hansen B. (2011). GDF-15 Is an Inhibitor of Leukocyte Integrin Activation Required for Survival after Myocardial Infarction in Mice. Nat. Med..

[9] Lok S.I., Winkens B., Goldschmeding R., van Geffen A.J.P., Nous F.M.A., van Kuik J., van der Weide P., Klöpping C., Kirkels J.H., Lahpor J.R. (2012). Circulating Growth Differentiation Factor-15 Correlates with Myocardial Fibrosis in Patients with Non-Ischaemic Dilated Cardiomyopathy and Decreases Rapidly after Left Ventricular Assist Device Support. Eur. J. Heart Fail..

[10] Ahmad T., Wang T., O’Brien E.C., Samsky M.D., Pura J.A., Lokhnygina Y., Rogers J.G., Hernandez A.F., Craig D., Bowles D.E. (2015). Effects of Left Ventricular Assist Device Support on Biomarkers of Cardiovascular Stress, Fibrosis, Fluid Homeostasis, Inflammation, and Renal Injury. JACC Heart Fail..

[11] Rossaint J., Vestweber D., Zarbock A. (2013). GDF-15 Prevents Platelet Integrin Activation and Thrombus Formation. J. Thromb. Haemost..

[12] Lippi G., Salvagno G.L., Danese E., Brocco G., Gelati M., Montagnana M., Sanchis-Gomar F., Favaloro E.J. (2017). Serum Concentration of Growth Differentiation Factor-15 Is Independently Associated with Global Platelet Function and Higher Fibrinogen Values in Adult Healthy Subjects. Semin. Thromb. Hemost..

[13] Arauna D., García F., Rodríguez-Mañas L., Marrugat J., Sáez C., Alarcón M., Wehinger S., Espinosa-Parrilla Y., Palomo I., Fuentes E. (2020). Older Adults with Frailty Syndrome Present an Altered Platelet Function and an Increased Level of Circulating Oxidative Stress and Mitochondrial Dysfunction Biomarker GDF-15. Free Radic. Biol. Med..

[14] Wallentin L., Hijazi Z., Andersson U., Alexander J.H., De Caterina R., Hanna M., Horowitz J.D., Hylek E.M., Lopes R.D., Åsberg S. (2014). Growth Differentiation Factor 15, a Marker of Oxidative Stress and Inflammation, for Risk Assessment in Patients With Atrial Fibrillation. Circulation.

[15] Hagström E., James S.K., Bertilsson M., Becker R.C., Himmelmann A., Husted S., Katus H.A., Steg P.G., Storey R.F., Siegbahn A. (2016). Growth Differentiation Factor-15 Level Predicts Major Bleeding and Cardiovascular Events in Patients with Acute Coronary Syndromes: Results from the PLATO Study. Eur. Heart J..

[16] Berg D.D., Ruff C.T., Jarolim P., Giugliano R.P., Nordio F., Lanz H.J., Mercuri M.F., Antman E.M., Braunwald E., Morrow D.A. (2019). Performance of the ABC Scores for Assessing the Risk of Stroke or Systemic Embolism and Bleeding in Patients With Atrial Fibrillation in ENGAGE AF-TIMI 48. Circulation.

[17] Gremmel T., Michelson A.D., Frelinger A.L., Bhatt D.L. (2018). Novel Aspects of Antiplatelet Therapy in Cardiovascular Disease. Res. Pract. Thromb. Haemost..

[18] Tscharre M., Michelson A.D., Gremmel T. (2020). Novel Antiplatelet Agents in Cardiovascular Disease. J. Cardiovasc. Pharmacol. Ther..

[19] Gremmel T., Steiner S., Seidinger D., Koppensteiner R., Panzer S., Kopp C.W. (2014). In Vivo and Protease-Activated Receptor-1-Mediated Platelet Activation but Not Response to Antiplatelet Therapy Predict Two-Year Outcomes after Peripheral Angioplasty with Stent Implantation. Thromb. Haemost..

[20] Michelson A.D., Barnard M.R., Krueger L.A., Valeri C.R., Furman M.I. (2001). Circulating Monocyte-Platelet Aggregates Are a More Sensitive Marker of in Vivo Platelet Activation than Platelet Surface P-Selectin: Studies in Baboons, Human Coronary Intervention, and Human Acute Myocardial Infarction. Circulation.

[21] Furman M.I., Benoit S.E., Barnard M.R., Valeri C.R., Borbone M.L., Becker R.C., Hechtman H.B., Michelson A.D. (1998). Increased Platelet Reactivity and Circulating Monocyte-Platelet Aggregates in Patients with Stable Coronary Artery Disease. J. Am. Coll. Cardiol..

[22] Shattil S.J., Hoxie J.A., Cunningham M., Brass L.F. (1985). Changes in the Platelet Membrane Glycoprotein IIb.IIIa Complex during Platelet Activation. J. Biol. Chem..

[23] Tóth O., Calatzis A., Penz S., Losonczy H., Siess W. (2006). Multiple Electrode Aggregometry: A New Device to Measure Platelet Aggregation in Whole Blood. Thromb. Haemost..

[24] Sibbing D., Aradi D., Alexopoulos D., ten Berg J., Bhatt D.L., Bonello L., Collet J.-P., Cuisset T., Franchi F., Gross L. (2019). Updated Expert Consensus Statement on Platelet Function and Genetic Testing for Guiding P2Y12 Receptor Inhibitor Treatment in Percutaneous Coronary Intervention. JACC Cardiovasc. Interv..

[25] Mayer K., Bernlochner I., Braun S., Schulz S., Orban M., Morath T., Cala L., Hoppmann P., Schunkert H., Laugwitz K.-L. (2014). Aspirin Treatment and Outcomes After Percutaneous Coronary Intervention. J. Am. Coll. Cardiol..

[26] Sibbing D., Braun S., Morath T., Mehilli J., Vogt W., Schömig A., Kastrati A., von Beckerath N. (2009). Platelet Reactivity after Clopidogrel Treatment Assessed with Point-of-Care Analysis and Early Drug-Eluting Stent Thrombosis. J. Am. Coll. Cardiol..

[27] Sibbing D., Schulz S., Braun S., Morath T., Stegherr J., Mehilli J., Schömig A., Von Beckerath N., Kastrati A. (2010). Antiplatelet Effects of Clopidogrel and Bleeding in Patients Undergoing Coronary Stent Placement. J. Thromb. Haemost..

[28] Michelson A.D. (2010). Antiplatelet Therapies for the Treatment of Cardiovascular Disease. Nat. Rev. Drug Discov..

[29] de Bruyn K.M.T., Zwartkruis F.J.T., de Rooij J., Akkerman J.-W.N., Bos J.L. (2003). The Small GTPase Rap1 Is Activated by Turbulence and Is Involved in Integrin AIIbβ3-Mediated Cell Adhesion in Human Megakaryocytes. J. Biol. Chem..

[30] Lindholm D., Hagström E., James S.K., Becker R.C., Cannon C.P., Himmelmann A., Katus H.A., Maurer G., López-Sendón J.L., Steg P.G. (2017). Growth Differentiation Factor 15 at 1 Month After an Acute Coronary Syndrome Is Associated with Increased Risk of Major Bleeding. J. Am. Heart Assoc..

[31] Martorell L., Martínez-González J., Rodríguez C., Gentile M., Calvayrac O., Badimon L. (2008). Thrombin and Protease-Activated Receptors (PARs) in Atherothrombosis. Thromb. Haemost..

[32] Gremmel T., Eslam R.B., Koppensteiner R., Lang I.M., Panzer S. (2013). Prasugrel Reduces Agonists’ Inducible Platelet Activation and Leukocyte-Platelet Interaction More Efficiently than Clopidogrel. Cardiovasc. Ther..

[33] Wadowski P.P., Pultar J., Weikert C., Eichelberger B., Panzer B., Huber K., Lang I.M., Koppensteiner R., Panzer S., Gremmel T. (2019). Protease-Activated Receptor-Mediated Platelet Aggregation in Acute Coronary Syndrome Patients on Potent P2Y 12 Inhibitors. Res. Pract. Thromb. Haemost..

[34] Tricoci P., Huang Z., Held C., Moliterno D.J., Armstrong P.W., Van De Werf F., White H.D., Aylward P.E., Wallentin L., Chen E. (2012). Thrombin-Receptor Antagonist Vorapaxar in Acute Coronary Syndromes. N. Engl. J. Med..

[35] Pulcinelli F.M., Pignatelli P., Celestini A., Riondino S., Gazzaniga P.P., Violi F. (2004). Inhibition of Platelet Aggregation by Aspirin Progressively Decreases in Long-Term Treated Patients. J. Am. Coll. Cardiol..

[36] Hijazi Z., Oldgren J., Lindbäck J., Alexander J.H., Connolly S.J., Eikelboom J.W., Ezekowitz M.D., Held C., Hylek E.M., Lopes R.D. (2016). The Novel Biomarker-Based ABC (Age, Biomarkers, Clinical History)-Bleeding Risk Score for Patients with Atrial Fibrillation: A Derivation and Validation Study. Lancet.

[37] Song L., Söderholm M., Svensson E.H., Borné Y., Engström G. (2021). Circulating Growth Differentiation Factor 15 Levels Are Associated With Risk of Both Intracerebral and Subarachnoid Hemorrhage. Front. Neurol..

[38] Patrono C., García Rodríguez L.A., Landolfi R., Baigent C. (2005). Low-Dose Aspirin for the Prevention of Atherothrombosis. N. Engl. J. Med..

[39] Santilli F., Rocca B., De Cristofaro R., Lattanzio S., Pietrangelo L., Habib A., Pettinella C., Recchiuti A., Ferrante E., Ciabattoni G. (2009). Platelet Cyclooxygenase Inhibition by Low-Dose Aspirin Is Not Reflected Consistently by Platelet Function Assays. J. Am. Coll. Cardiol..

[40] Tscharre M., Wittmann F., Kitzmantl D., Lee S., Eichelberger B., Wadowski P.P., Laufer G., Wiedemann D., Forstner-Bergauer B., Ay C. (2021). Platelet Activation and Aggregation in Different Centrifugal-Flow Left Ventricular Assist Devices. Platelets.

[41] Schulman S., Kearon C. (2005). Definition of Major Bleeding in Clinical Investigations of Antihemostatic Medicinal Products in Non-Surgical Patients. J. Thromb. Haemost..

