# Supplementary material for: Growth Differentiation Factor-15 Correlates Inversely with Protease-Activated Receptor-1-Mediated Platelet Reactivity in Patients with Left Ventricular Assist Devices
Source: Pharmaceuticals (Basel). 2022 Apr 15;15(4):484. doi: 10.3390/ph15040484 (PMC9031879; doi:10.3390/ph15040484)
Supplement: Supplementary file 1 [file pharmaceuticals-15-00484-s001.zip › pharmaceuticals-1661567-supplementary.pdf]

Supplementary Table S1: Baseline characteristics comparing patients included in the final analysis and patients without available plasma sample.

	**Sample available**	**No Sample**	**p-value**
	N=51	***N=16***	
Age, years	62 [55-69]	62 [55-72]	0.612
Sex, No.(%):			
Female patients	5 (9.8)	1 (6.3)	0.664
Ventricular assist device, No.(%):			
HVAD	15 (29.4)	5 (31.3)	
HM2	2 (3.9)	1 (6.3)	0.908
HM3	34 (66.7)	10 (62.5)	
Body mass index, kg/m2	29.4 [25.9-31.8]	28.2 [26.3-29.3]	0.543
Type of cardiomyopathy, No.(%):			
ischemic CMP	38 (74.5)	11 (68.8)	0.650
dilatative CMP	13 (25.5)	5 (31.2)	
Flow, min-1	4.9 [4.3-5.2]	5.0 [3.8-5.1]	0.949
Speed RPM	5350 [2925-5700]	5550 [2800-5775]	0.595
History of arterial hypertension, No.(%)	29 (56.9)	8 (50)	0.630
Hyperlipidemia, No.(%)	23 (45.1)	10 (62.5)	0.224
Diabetes	10 (19.6)	7 (43.8)	0.053
Atrial fibrillation, No.(%)	20 (39.2%)	5 (31.3)	0.565
Cerebrovascular disease, No.(%)	2 (3.9%)	0 (0.0)	0.701
Hemoglobin, g/dl	13.3 [11.7-14.4]	12.3 [11.5-13.7]	0.233
Thrombocytes, G/l	222 [178-265]	205 [170-299]	0.977
White blood cell count, G/l	8.28 [6.25-9.73]	8.7 [6.3-12.8]	0.410
Creatinine, mg/dl	1.18 [1.02-1.64]	1.6 [1.1-2.1]	0.061
INR	2.40 [2.10-2.70]	2.4 [1.9-2.6]	0.412

	Sample available	No Sample	p-value
	N=51	***N=16***	
proBNP	1022 [567-1691]	2708 [1084-3951]	0.015
high sensitivity C-reactive protein, mg/l	0.27 [0.13-0.59]	0.47 [0.34-1.44]	0.056
Vitamin K antagonist, No.(%)	51 (100%)	16 (100%)	.
Aspirin, No.(%)	51 (100%)	16 (100%)	.
ACE-I or ARB, No.(%)	36 (70.6%)	6 (37.5)	0.054
Furosemide, No.(%)	45 (88.2%)	13 (81.3)	0.475
Statin, No.(%)	34 (68.0%)	12 (80)	0.424
